# The common cold: The need for an effective treatment amid the FDA discussion on oral phenylephrine

**DOI:** 10.1016/j.jacig.2024.100318

**Published:** 2024-08-07

**Authors:** Emina Išerić, Joris C. Verster

**Affiliations:** a Division of Pharmacology, Utrecht Institute for Pharmaceutical Sciences, Utrecht University, Utrecht, The Netherlands; b Centre for Mental Health and Brain Sciences, Swinburne University, Melbourne, Australia; c Cognitive Neurophysiology, Department of Child and Adolescent Psychiatry, Faculty of Medicine, TU Dresden, Dresden, Germany

**Keywords:** Rhinovirus, upper respiratory tract infection, SJP-002, NSAID, antihistamine, decongestant, mast cell stabilizer, phenylephrine

## Abstract

An episode of the common cold can have a significant negative impact on quality of life, mood, and daily activities. In line with this fact, there is a growing demand for health care and treatments associated with the common cold. Current treatments aim to (1) inhibit symptom severity and (2) shorten the duration of an episode of the common cold. These products include analgesics, antihistamines, and decongestants. In addition, various supplements, including vitamins, minerals, and herbs, are marketed to treat the common cold. The current products marketed for treating the common cold may reduce the severity of some (but not all) common cold symptoms, but they usually do not shorten the common cold episode. The recent indication that phenylephrine is not effective means that it will ultimately need to be removed from the over-the-counter monograph. Manufacturers will consequently need to reformulate their products and withdraw oral phenylephrine-containing products. Several newly developed common cold products are currently under investigation. These clinical trials should evaluate their efficacy and safety, as there remains a clear need for common cold products that significantly reduce both the symptom severity and the duration of episodes of the common cold.

Upper respiratory tract infection, often referred to as the common cold, is characterized by various symptoms such as sore throat, nasal congestion, coughing, sneezing, and body aches. In most cases, the common cold is caused by a rhinovirus.^1^^,^^2^ According to the Global Burden of Diseases, Injuries, and Risk Factors Study, in 2019, there were 17.2 billion cases of the common cold worldwide.^3^ As such, the common cold accounted for almost 50% of all cases of burden of disease.^3^ Depending on the individual’s health status, the duration of a common cold episode can be shorter or longer. In healthy individuals, a common cold episode is usually resolved within 1 to 2 weeks. However, for individuals with immune-related conditions (eg, patients with asthma or bronchitis), up to 3 weeks or more may be required to fully recover from a common cold episode. During this period of reduced immune fitness, such individuals have an increased susceptibility to develop more severe illnesses, such as pneumonia.^1^^,^^2^

Experiencing common cold symptoms can have a significant negative impact on skills and abilities that are critical to performing daily activities (eg, driving a car)^4^ and mood. Together, the effects may have a significant negative influence on quality of life.^5^^,^^6^ The economic impact of the common cold is evident from the fact that it is the primary cause of not going to work (absenteeism) and attending work while feeling ill (presenteeism). Presenteeism on days when a worker is experiencing a common cold is associated with significantly reduced productivity and poorer work performance.^7^ Surprisingly, there are few recent data available on the related costs for the economy. A US study conducted more than 20 years ago estimated that the common cold was associated with approximately 70 to 120 million lost workdays per year.^7^ The annual costs of absenteeism and presenteeism due to the common cold were estimated to be between $25 billion and $40 billion.^7^ Next to the direct costs to the economy, the common cold is also associated with significant demands on the health care system from the standpoints of physician visits and prescription of treatments.

Overall, the market for cold and cough products is expected to grow significantly.^8^ In 2016, sales totaled $29.2 billion, whereas in 2022, they had increased to $39.26 billion. The United States (in which sales totaled $10.21 billion) and China (in which sales totaled $9.01 billion) accounted for about half of the market revenues. For the next 5 years, a further annual increase of the common cold market by 5.6% is forecast, with sales in 2027 projected to total $53.1 billion. The most substantial growth (8%) is expected in China, but with an average per capita revenue of $30.50, the United States remains the biggest market.^8^ Together, these data show that there is an increasing demand for common cold products.

Given the common cold’s negative impact on daily life and health, various methods are applied to prevent common cold infection and treat the common cold once a person has been infected. The ideal treatment for the common cold would reduce both its severity and its duration of (Fig 1).^9^

**Fig 1:**
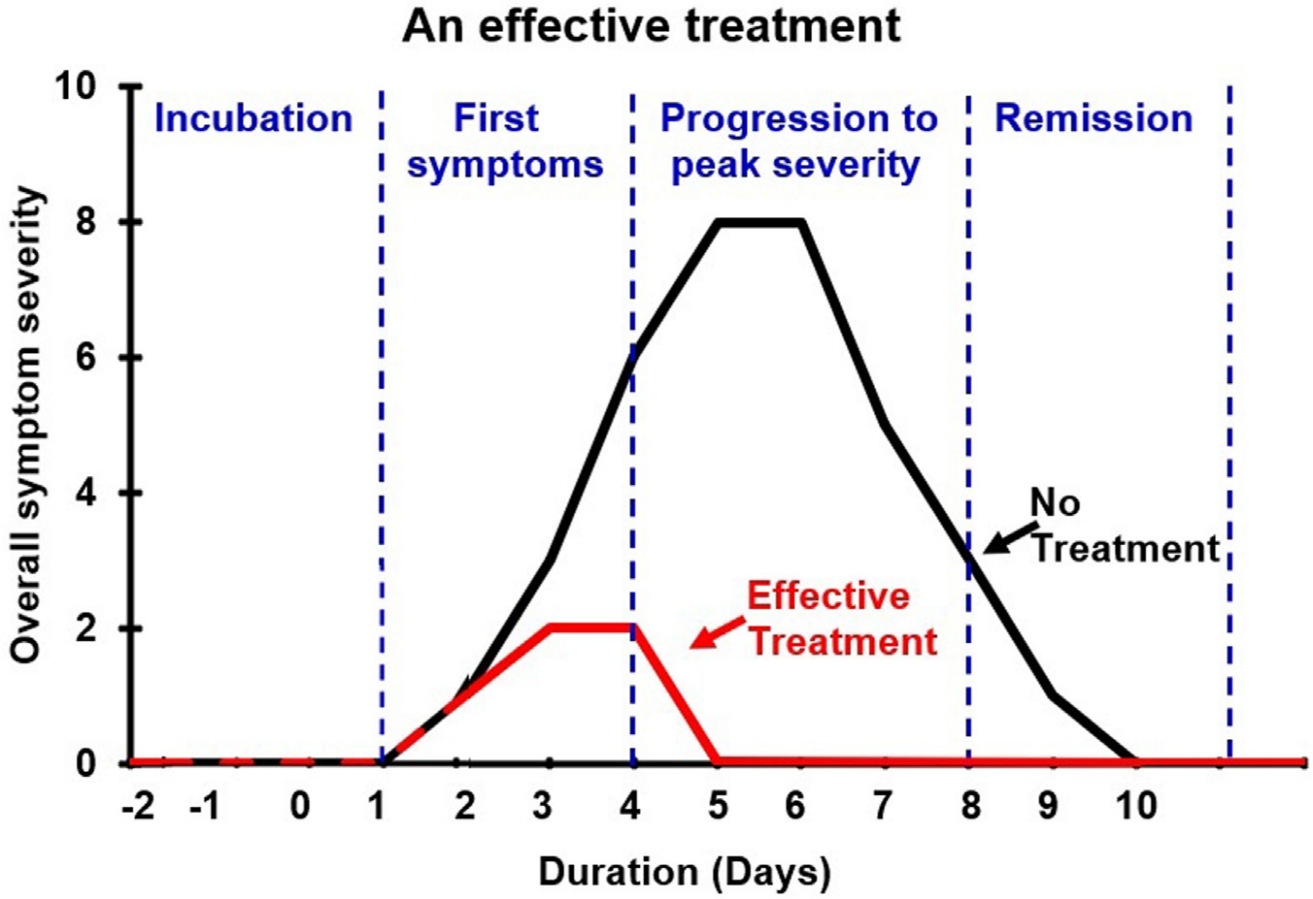
Schematic representation of the effects of an effective common cold treatment. After being infected, individuals enter the incubation phase (day –2 to day 0), followed by the first appearance of symptoms (days 1-3), a progression of symptoms to peak severity (days 4-7), and a remission phase during which symptoms resolve (days 8-10).^9^ In this example, a 75% reduction in peak overall symptom severity and 50% reduction in the duration of the common cold episode are shown.

Nonpharmacologic measures to prevent the common cold include using face masks and practicing regular hand washing and disinfection. However, a Cochrane review concluded that wearing a medical mask made little to no difference compared with not wearing a mask, and an N95 mask (ie, a mask that it filters at least 95% of airborne particles having a mass median aerodynamic diameter of 0.3 μm) had no benefit over a medical/surgical mask.^10^ Studies show that proper hand hygiene does reduce the infection rate by 11%. Once an individual has been infected, steaming (inhaling heated water vapor) is another commonly suggested method to alleviate common cold symptoms. However, the literature shows that steaming is not able to reduce common cold symptom severity, and it does not shorten the duration of the common cold.^11^

The majority of individuals self-administer over-the-counter (OTC) cough and cold products to combat the common cold. Pharmacologic interventions to treat the common cold include analgesics, antihistamines, decongestants, and a combination of these medications.^2^ These treatments aim to alleviate the symptoms of a common cold, and several studies have demonstrated the (partial) efficacy of these products. However, the disadvantage of these products is that they reduce only some but not all of the symptoms of the common cold. For instance, acetaminophen (paracetamol) may improve nasal obstruction and runny nose, but it shows no significant improvement in other symptoms, such as sore throat, coughing or general malaise.^12^ Other popular treatments include vitamin supplements, herbal products, and honey. However, review studies have revealed only low- to moderate-quality evidence to support the efficacy of these products in reducing severity, with no impact on duration of the common cold episode.^13^ Alternatively, zinc supplementation did not reduce the severity of the common cold episode, although it has been shown to reduce the duration of a common cold episode. However, the use of zinc may be associated with bad taste and nausea. Therefore, individuals who want to use zinc should weigh the benefit of a potential shortening of the common cold episode against the risk of these unwanted side effects.^14^ Finally, a Cochrane review concluded that use of intranasal corticosteroids for symptomatic relief from the common cold was not effective.^15^ Taken together, the data indicate that currently, there is no treatment available for which scientific research has demonstrated ability to reduce both the severity and the duration of a common cold episode.

An OTC product that has recently gained attention is the oral decongestant phenylephrine. Phenylephrine has been listed in the Cold, Cough, Allergy, Bronchodilator, and Antiasthmatic (CCABA) OTC monograph since 1976.^16^ Being in the CCABA OTC monograph means that that these drugs are generally recognized as safe and effective and are available as nonprescription medication. Oral phenylephrine has been used since the 1970s, with a significant increase in sales from 2006 as a pseudoephedrine alternative owing to restrictions on the use of pseudoephedrine in an effort to combat the methamphetamine epidemic.^17^ Recently, the US Food and Drug Administration’s Nonprescription Advisory Committee reevaluated the efficacy of oral phenylephrine and deemed the drug ineffective in relieving the symptoms of nasal congestion at the recommended dosage.^18^ Although the committee considers the recommended dose (10 mg every 4 hours) safe, the indication that it is not effective means that it will ultimately need to be removed from the OTC monograph. Manufacturers would consequently need to reformulate their products and withdraw oral phenylephrine-containing products from US retail and nonretail counters.

The impact of a withdraw of oral phenylephrine-containing products is evident. In 2022, the US retail market saw more than 240 million sales of oral phenylephrine-containing products, which is almost 5 times more than sales of products containing oral pseudoephedrine, reaching $1.76 billion in revenue, which was a 45% economic increase compared with the amount in 2021.^18^ Pseudoephedrine and phenylephrine are the only oral decongestants listed in the CCABA OTC monograph. Given the restricted use of pseudoephedrine and the potential withdrawal of phenylephrine, there is an urgent need for new effective and safe alternative treatments for the common cold.

A search on the website www.ClinicalTrials.gov and PubMed (on December 20, 2023) revealed a number of potential new common cold treatments that are currently in development. Some of these include new pathophysiologic approaches to combat the common cold, such as seawater-based formulations (ClinicalTrials.gov identifiers NCT05244148, NCT05034328, and NCT05365789), a combination of *Bifidobacterium breve* LA 708, extract of cypress *Cupressus sempervirens* L, extract of *Glycyrrhiza glabra* L (of the family Leguminosae), vapendavir (NCT06149494), XC221 (NCT05030324), omalizumab (NCT05332067), ingavirin (NCT03154515 and NCT05269290), Healsea Rescue (Lallemand, Montréal, Québec, Canada) nasal spray with a natural Symbiofilm (Lallemand) extract isolated from the marine bacteria (NCT05819190), 2-deoxyglucose (NCT05314933), the herbal medicines Eungyosan and Samsoeum (NCT04073511), and many more. These products may exert alternative mechanisms of action that should be further investigated, as this will further increase our understanding of the pathology of the common cold. Lastly, a recently published case report^19^ investigated the efficacy of SJP-002, a combination of the nonsteroidal anti-inflammatory drug naproxen and the antihistamine drug fexofenadine. Individuals who self-administered SJP-002 showed a significant reduction in both severity of symptoms and duration of the common cold episode.^19^ Regarding the mechanism of action of SJP-002, it is proposed that the combined anti-inflammatory action of the nonsteroidal anti-inflammatory drug and the mast cell–stabilizing effect of the antihistamine drug if taken when the first common cold symptoms occur will limit symptom severity and duration before progression into the more severe active phase of the common cold (Fig 1). Although the case report showed promising results, SJP-002 also needs to be further investigated in double-blind, placebo-controlled trials. When they become available, the results from the clinical trials of these drugs under investigation will provide a better understanding of the pathology of the common cold and which treatment approach is most effective.

Taken together, the data confirm the urgent need for an effective and safe treatment for the common cold that can both alleviate symptom severity and shorten the duration and of a common cold episode. The bar for new common cold treatments is very high, as new OTC treatments need to be effective, safe, and (almost) devoid of side effects.

## Disclosure statement

Disclosure of potential conflict of interest: Over the past 36 months, J. C. Verster has acted as a consultant and/or expert advisor to Eisai, KNMP, Med Solutions, Red Bull, Sen-Jam Pharmaceutical, and Toast!, and he owns stock in and has received travel support from Sen-Jam Pharmaceutical. Over the past 36 months, E. Išerić has received travel support from Sen-Jam Pharmaceutical.
